# Impact of hand function impairment on daily life of patients with systemic sclerosis: a qualitative study

**DOI:** 10.1093/rheumatology/keaf476

**Published:** 2025-09-09

**Authors:** Mark Greveling, Stefano Rodolfi, Nora El Bardai, Christopher P Denton, Voon H Ong, Nick Jeffries-Owen, Rita Schriemer, Lieke Tweehuysen, Julia Spierings

**Affiliations:** Department of Rheumatology & Clinical Immunology, University Medical Centre Utrecht, Utrecht, The Netherlands; Division of Medicine, Department of Inflammation, Centre for Rheumatology and Connective Tissue Diseases, Royal Free Hospital and University College London, London, UK; Department of Rheumatology & Clinical Immunology, University Medical Centre Utrecht, Utrecht, The Netherlands; Division of Medicine, Department of Inflammation, Centre for Rheumatology and Connective Tissue Diseases, Royal Free Hospital and University College London, London, UK; Division of Medicine, Department of Inflammation, Centre for Rheumatology and Connective Tissue Diseases, Royal Free Hospital and University College London, London, UK; Scleroderma & Raynaud’s UK (SRUK), London, United Kingdom; National Association for People with Lupus, Systemic Sclerosis, Antiphospholipid Syndrome, and Mixed Connective Tissue Disease (NVLE), Utrecht, The Netherlands; Department of Rheumatology & Clinical Immunology, University Medical Centre Utrecht, Utrecht, The Netherlands; Department of Rheumatology & Clinical Immunology, University Medical Centre Utrecht, Utrecht, The Netherlands; Division of Medicine, Department of Inflammation, Centre for Rheumatology and Connective Tissue Diseases, Royal Free Hospital and University College London, London, UK

**Keywords:** systemic sclerosis, scleroderma, interview studies, qualitative research, hand function impairment

## Abstract

**Objectives:**

Many patients with systemic sclerosis (SSc) experience impaired hand function, yet the precise nature and impact of this impairment remains unclear. In this study, we explored the determinants of hand function impairment in SSc from a patient perspective and its impact on daily life. Additionally, we identified unmet care needs related to hand function impairment.

**Methods:**

Adult patients with SSc were included from the University Medical Centre Utrecht, the Netherlands, and Royal Free Hospital London, United Kingdom (UK). Face-to-face semi-structured interviews were conducted, transcribed verbatim and coded. Thematic analysis was performed to identify key themes. Hand function was evaluated using the modified Hand Mobility in Scleroderma (mHAMIS) and the Cochin Hand Function Scale (CHFS).

**Results:**

Thirty-three patients were included (*n* = 18 in the Netherlands, *n* = 15 in the UK). Three main themes were identified: symptoms, impact and (un)met needs. The symptoms theme captures the broad range of medical and functional complaints, often co-occurring and leading to significant hand function impairment. The impact theme describes how these symptoms limited daily activities, employment and leisure, and contributed to emotional distress and social isolation. The (un)met needs theme highlights varied coping strategies and experiences with care. While participants felt that patient education was sufficient when healthcare professionals addressed hand impairment, many reported a lack of tailored support and insufficient recognition of hand-related problems.

**Conclusion:**

Hand function impairment in SSc profoundly affects patients’ daily lives and well-being. Addressing this unmet need requires greater clinical awareness and more personalised and symptom-specific management strategies.

Rheumatology key messages

- Hand function impairment is a key contributor to disability in systemic sclerosis, significantly affecting daily activities and overall quality of life.
- A personalised symptom-specific approach with regular monitoring is preferred from the patient’s perspective.
- A significant clinical need exists to understand the underlying mechanisms for hand function impairment.

## Introduction

Systemic sclerosis (SSc) is a rare autoimmune connective tissue disease characterised by widespread microvascular damage, skin thickening and fibrotic changes in organs [1]. Traditionally, research on SSc has primarily focused on organ damage and mortality, as pulmonary, cardiac and renal involvements are critical determinants of survival. However, non-lethal manifestations of SSc significantly impact patients’ quality of life and daily functioning. Among these, hand function impairment is particularly prevalent and has profound consequences for daily activities. Notably, it accounts for 75% of variance in global disability among SSc patients, making it a key contributor to overall disability [2, 3]. This impairment substantially affects activities of daily living and work participation, further contributing to poor quality of life [4, 5]. Many SSc patients identify hand-related tasks as their greatest challenge [6].

Despite its substantial burden, current standard-of-care treatments rarely improve hand function, which tends to worsen progressively over time [7, 8]. A Dutch cross-sectional study of 650 SSc patients found that 28% identified reduced hand function as an unmet care need, underscoring the pressing need for targeted and effective interventions [9].

Although this unmet need is recognised, in-depth understanding of patients’ perspectives regarding hand function impairment and their specific care needs remains limited. This gap hinders the development of effective management strategies. Therefore, this study seeks to investigate factors contributing to hand function impairment in SSc from the patients’ perspective, evaluate its effects on daily functioning and identify any unmet care needs.

## Methods

### Study design

This is a multicentre qualitative semi-structured interview study in the Netherlands and the United Kingdom. In the Netherlands, the Medical Ethics Review Committee determined that this study did not fall within the scope of the Medical Research Involving Human Subjects Act (WMO) and was therefore exempt from formal ethical approval (23U-0266). In the United Kingdom, this study was approved by HRA and Health Care Research Wales (HCRW) (23/EM/0288).

### Participants

Adult patients with established diagnosis of SSc and fulfilling the EULAR-ACR 2013 criteria, treated at the rheumatology departments of the University Medical Centre Utrecht (the Netherlands) and the Royal Free Hospital London (United Kingdom), were invited to participate [10]. Patients who did not speak Dutch (Netherlands) or English (UK) were excluded. Patients were invited consecutively during routine visits by their treating physician from June 2023 until October 2024. Patients were purposefully selected as heterogeneity was pursued concerning age, gender and disease duration. Thirty-five patients were approached, one patient was excluded because of a language barrier, one declined participation because the questions would be too distressing. Written informed consent was obtained before the interview. Interviews lasted ∼20–40 min. The number of interviews was determined by consensual agreement that analytical saturation was achieved, while considering the concept of information power [11, 12].

### Data collection

An interview guide with open-ended questions was used to facilitate in-depth, semi-structured, face-to-face interviews (Supplementary Data S1 for the Dutch interview guide, Supplementary Data S2 for the English interview guide). The original Dutch version of the guide was forward-backward translated into English by native speakers and pilot-tested in both languages. The interview guide was developed in collaboration with representatives of the Dutch and British patient organisations for SSc (NVLE and SRUK). These representatives reviewed the initial draft, provided feedback on the wording and relevance of the questions, and suggested additional topics related to work activities. Trained interviewers conducted the interviews at the hospital: N.E.B. (female medical student) in the Netherlands and S.R. (male MD, research fellow) in the UK. The interviews were audio recorded, transcribed verbatim and anonymised. After the initial interviews, researchers (M.G., L.T., J.S.) reviewed and discussed the transcripts to reflect on phrasing and sequencing of questions. Minor refinements were made to the interview approach to enhance richness and relevance of the data.

To provide contextual information on the level of impairment among participants, hand function was assessed with the modified Hand Mobility in Scleroderma (mHAMIS) functional range of motion test and Cochin Hand Function Scale (CHFS) questionnaire of patient-reported outcome measures. Hands were evaluated for digital ulcers, pitting scars, gangrene, arthritis, contractures, tendon friction rubs and puffy hands [13, 14]. Additionally, modified Rodnan Skin Score (mRSS), weight and length were collected.

### Data analysis

Data collection and analysis were performed simultaneously. Written transcripts were analysed using Nvivo (V15) within the framework of thematic content analysis [15]. Two researchers (M.G. and L.T.) independently performed open coding of the first three interviews from each country, followed by joint discussions to compare interpretations, resolve discrepancies and refine the initial coding framework. Codes were grouped into themes through an iterative and reflective process. After this initial phase, one researcher (M.G.) continued coding the remaining transcripts using the agreed framework, while regularly discussing emerging findings and interpretations with the wider research team. The patient partners were involved in data interpretation and drafting of the article.

All statistical analyses of quantitative data were performed using SPSS v25.0 (SPSS, Chicago, IL, USA). Results were reported following the Consolidated Criteria for Reporting Qualitative Research (COREQ) checklist (see Supplementary Data S3) [16].

## Results

In the Netherlands, 18 patients were included before analytical saturation was achieved. In the United Kingdom, 15 patients were included. Most participants were female (76%) and Caucasian (91%), with a median age of 60 years. Most patients had diffuse cutaneous SSc (55%), with a median disease duration of 8 years. The CHFS and mHAMIS scores indicated a moderate-to-high degree of hand function impairment. A complete summary of characteristics is shown in Table 1.

**Table 1.** Participant characteristics

	Overall (*n* = 33)	The Netherlands (*n* = 18)	United Kingdom (*n* = 15)
Demographics			
Female, *n* (%)	25 (76%)	16 (89%)	9 (60%)
Age, median (IQR)	60 (48–67)	61 (54–69)	53 (44–63)
Ethnicity			
Caucasian, *n* (%)	30 (91%)	16 (89%)	14 (93%)
Other, *n* (%)	3 (9%)	2 (11%)	1 (7%)
Employment status			
Fulltime job, *n* (%)	11 (33%)	5 (28%)	6 (40%)
Part-time job, *n* (%)	4 (12%)	2 (11%)	2 (13%)
Retired, *n* (%)	7 (21%)	3 (17%)	4 (27%)
Unemployed, *n* (%)	3 (9%)		3 (20%)
Work disabled, *n* (%)	6 (18%)	6 (33%)	
Part-time volunteer, *n* (%)	1 (3%)	1 (6%)	
Jobseeker, *n* (%)	1 (3%)	1 (6%)	
Disease characteristics			
Diagnosis			
dcSSc, *n* (%)	18 (55%)	7 (39%)	11 (73%)
lcSSc, *n* (%)	14 (42%)	10 (55%)	4 (27%)
Undifferentiated, *n* (%)	1 (3%)	1 (6%)	
Disease duration, median (IQR) years	8 (3–13)	4 (2–13)	8 (6–13)
mRSS score (0–51), median (IQR)	8 (4–16)	8 (5–16)^a^	6 (4–18)
mHAMIS score (0–18), median (IQR)	4 (2–8)	3 (2–5)	7 (3–11)
CHFS score (0–90), median (IQR)	32 (26–48)	36 (25–47)^a^	31 (26–62)^b^

a One score missing.

b Eight scores are missing.

CHFS: Cochin Hand Function Scale; dcSSc: diffuse cutaneous systemic sclerosis; IQR: interquartile range; lcSSc: limited cutaneous systemic sclerosis; mHAMIS: modified hand mobility in scleroderma; mRSS: modified Rodnan skin score.

**Figure 1.**
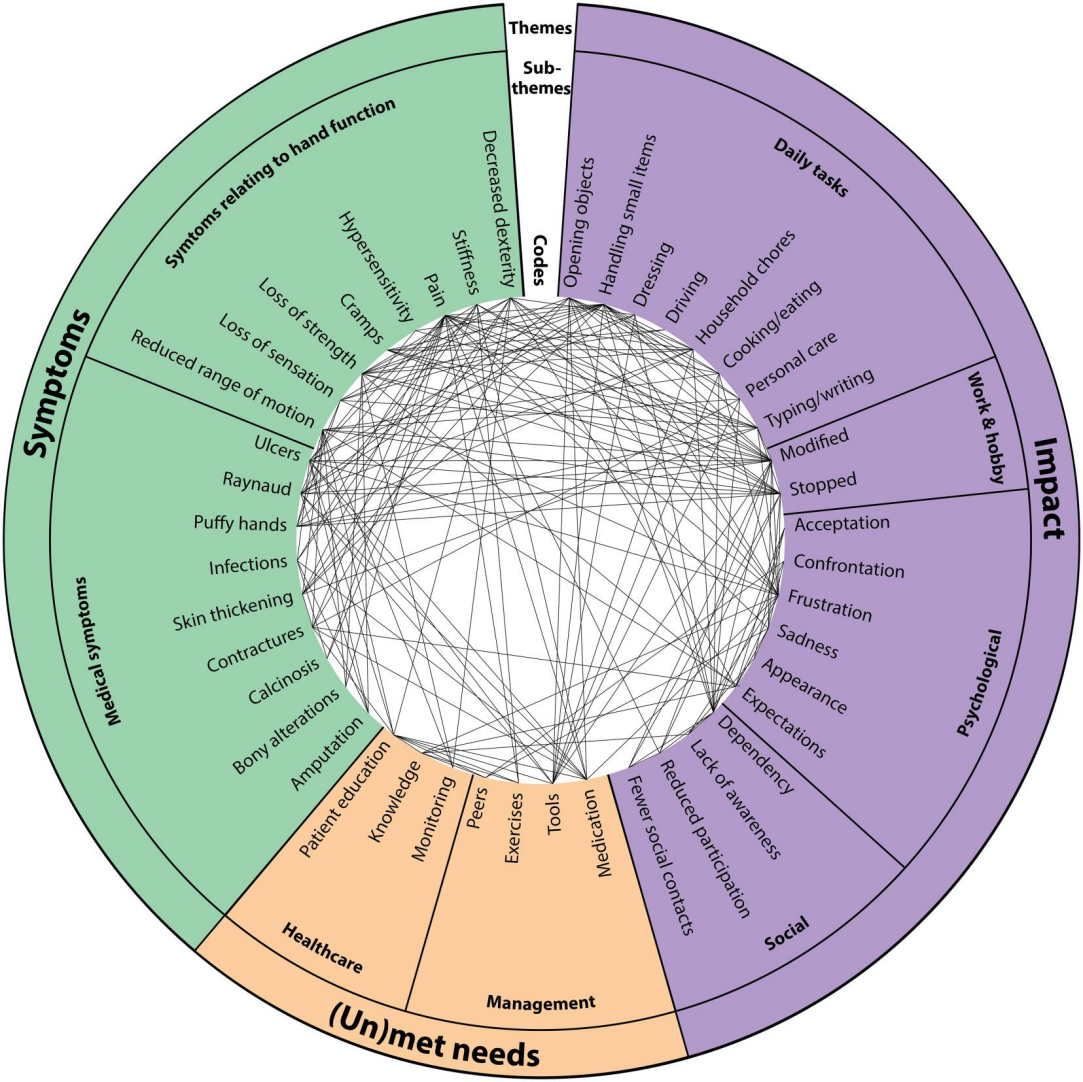
Overview of the themes, sub-themes and codes identified from the interviews, presented as a chord diagram. The outer ring represents the three main themes (Symptoms, Impact and (Un)met needs), subdivided into sub-themes and specific codes. Lines across the circle represent co-occurrence of codes within individual interviews, indicating how different symptoms, impacts and needs are interconnected in patients’ experiences. The density of connections illustrates the complexity and multidimensional nature of hand function impairment in SSc

Three main but interconnected themes were identified: symptoms, impact and (un)met needs. Each theme is further subdivided into sub-themes and codes. The symptom theme encompasses both medical and functional complaints, resulting in significant impairment of hand function. The impact theme describes how these symptoms limited activities, employment and leisure, and contributed to emotional distress and social isolation. The (un)met needs theme highlights varied coping strategies and experiences with care. As shown in Fig. 1, themes, sub-themes and individual codes were highly interconnected, reflecting the complex and multidimensional nature. It illustrates how symptoms, impacts and (un)met needs often co-occurred within patient experiences.

### Symptoms

**Figure 2.**
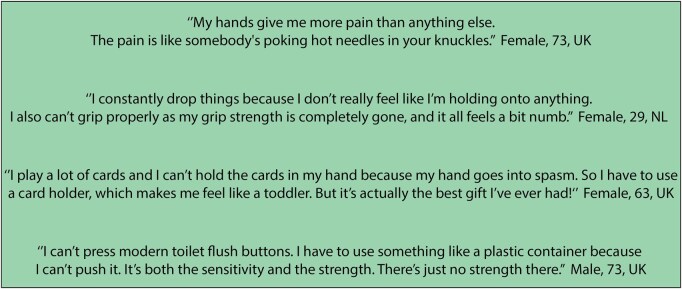
Quotes on symptoms regarding hand function impairment

Participants described various symptoms contributing to hand function impairment, which were categorised into medical symptoms (clinical manifestations or medical diagnoses) and symptoms relating to hand function (participants’ subjective experiences). Fig. 2 provides illustrative quotes. Many participants reported that the initial onset of disease manifested through symptoms in their hands, such as Raynaud’s, stiffness, loss of sensation and puffy hands.

#### Medical symptoms

Participants reported several medical symptoms, including Raynaud’s, ulcers, amputation, puffy hands, skin thickening, contractures, infections, calcinosis and bony alterations.

These medical symptoms were linked to various functional complaints. For instance, Raynaud’s, often triggered by cold environments or occasionally stress, was associated with pain, tingling and loss of sensation. Ulcers were reported as particularly difficult due to their slow healing process, pain, hypersensitivity and infection risk. Some participants felt stress could worsen wound formation. Amputation, typically resulting from blood flow restrictions or infections, led to losing strength and functionality. Puffy hands were often connected to temperature changes, stress or heavy labour and were associated with stiffness, reduced range of motion (ROM) and loss of sensation. Skin thickening and contractures were mainly linked to pain and restricted movement. Infections, frequently perceived to be triggered by stress resulted in stiffness and pain. Calcinosis was similarly associated with infections, pain and stiffness. Participants also observed changes in the shape of their hands due to bony alterations or arthritis, both linked to pain, loss of strength and reduced ROM.

#### Symptoms related to hand function

Participants most frequently reported symptoms such as pain, stiffness, loss of strength and sensation, decreased dexterity, cramps, hypersensitivity and reduced ROM.

Pain, observed throughout the entire hand but mainly the fingertips, was often linked to repetitive activities, bumping and exposure to cold. Some participants noted that pain disrupted sleep. Stiffness was experienced mainly in the morning but could also occur later in the day, in cold environments or after extensive activity. Loss of strength was commonly reported and often related to stiffness. Loss of sensation, usually affecting the fingertips or, occasionally, the entire hand, was frequently triggered by cold environments. This symptom led to difficulty perceiving tactile input, impairing functionality. A combination of reduced strength and sensation significantly contributed to decreased dexterity. Cramps were noted during extensive activities, while hypersensitivity, especially in the fingertips, was exacerbated by bumping or exposure to cold. The reduced ROM was primarily caused by inability to fully extend or spread the fingers, limiting overall hand span and mobility.

### Impact

**Figure 3.**
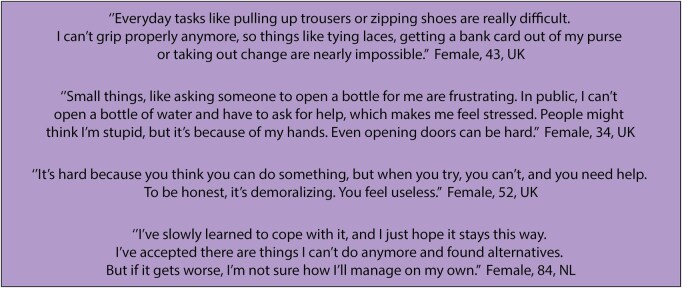
Quotes on the impact of hand function impairment

Hand function impairment affected various aspects of daily life, including daily tasks, work and hobbies, and had psychological and social implications. Fig. 3 provides illustrative quotes.

#### Daily tasks

Participants reported significant difficulties with daily tasks, including opening objects, handling small items, dressing, driving, household chores, cooking and eating, personal care, and typing and writing.

Opening objects such as bottles, jars, cans, containers, taps and doors was often challenging, as was using keys or extracting medication from packaging. These difficulties were attributed to lack of dexterity, strength, sensation and a reduced ROM. Handling small items, such as retrieving coins or cards from a wallet or bag, was similarly complex. Dressing presented challenges due to the intricate and precise movements required for buttons, zippers, laces, jewellery and socks, which many participants found almost impossible. Driving was considered manageable only with an automatic car, as shifting gears and operating the parking brake were difficult or impossible for some. Holding the steering wheel for extended periods was also challenging for some. Household chores, such as laundry, doing the dishes and cleaning were described as physically demanding due to pain, reduced grip strength and fatigue. Participants noted that tasks requiring repetitive movements or sustained pressure, like scrubbing or wringing, were particularly challenging. Cooking and eating were similarly affected, with difficulties reported in gripping cutlery, handling kitchen tools, lifting pans and opening packaging. Personal care was also significantly impacted. Activities such as washing, brushing teeth, styling hair or opening containers of shampoo were hindered by difficulty gripping objects or reaching specific areas. Typing and writing were difficult because participants could not feel what they were doing, experienced pain when pressing keys or found their actions less accurate. Touchscreens often failed to respond, compounding the challenge, while holding a pen was described as impossible for many. Tasks became especially difficult after prolonged use of the hands or in cold environments.

#### Work and hobbies

Hand function impairment significantly impacted work and hobbies, often necessitating modifications or even cessation. Many participants were forced to adapt work routines or hobbies. Typing and writing were particularly problematic, further limiting their ability to perform professional tasks or engage in hobbies requiring fine motor skills. Participants generally perceived support from company doctors positively. However, many individuals were incapacitated by their hand limitations, resulting in periods of absence or the need to leave their jobs entirely. Some participants switched to occupations that required less manual dexterity or hand involvement. Hobbies, including sports, gardening, sewing and playing musical instruments were also heavily impacted, forcing participants to stop or significantly adapt participation.

#### Psychological impact

The psychological effects of hand function impairment included difficulty with acceptance, confronting limitations, frustration, sadness, concerns about appearance and expectations.

Many patients reported struggling to accept their limitations, particularly in the early stages of disease. Over time, many learned to adapt and find ways to work around challenges. However, ongoing confrontation with physical limitations remained emotionally taxing, often leading to frustration and sadness. This frustration was heightened by difficulties performing daily tasks, inability to engage in hobbies, inability to work and perceived lack of understanding from others. Feelings of sadness or depression were frequently linked to persistent pain, inability to participate in activities, work or hobbies they enjoyed and dissatisfaction with the appearance of their hands. While changes in hand appearance were not troubling for most participants, some expressed shame or embarrassment and avoided showing their hands in public, describing them as unattractive. Additionally, some noted the inability to wear jewellery as a source of sadness and reminder of limitations. Regarding the future, many patients expressed hope that their hand function remains stable, though most feared further deterioration. The unpredictable course of the disease contributed to feelings of anxiety.

#### Social impact

Hand function impairment significantly impacted patients’ social interactions, leading to increased dependence, lack of understanding, reduced participation in activities and fewer social connections.

Many patients reported relying heavily on their partners, children, family, friends or even strangers for help with daily activities such as opening objects, completing household tasks, cooking, activities requiring dexterity, and hobbies. This dependence was often described as confronting and emotionally challenging, leading to frustration and sadness. These emotions were further exacerbated by lack of awareness from others about their hand function limitations. Patients expressed that others often failed to understand why they could not perform specific tasks, as their physical limitations were not always apparent. This frequently led to need for repeated explanations, described as tiring and emotionally draining. Inability to participate in social activities or hobbies due to hand limitations or cold environments further restricted patients’ social lives. This reduction in participation contributed to a sense of isolation and fewer social contacts. Additionally, greeting others by shaking hands was described as particularly painful, causing some patients to avoid this gesture altogether.

### (Un)met needs

**Figure 4.**
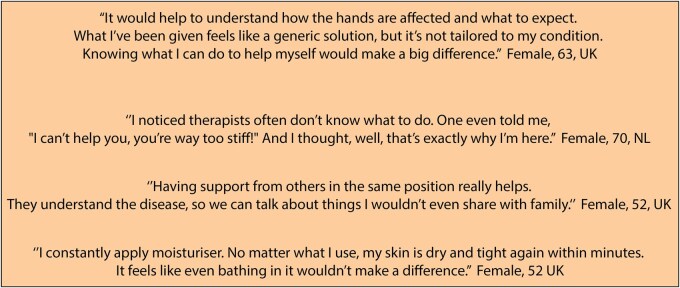
Quotes on (un)met needs regarding hand function impairment

Patients reported met and unmet needs regarding healthcare and management of hand function complaints. Fig. 4 provides illustrative quotes.

#### Healthcare

Patients highlighted positive aspects and areas for improvement in patient education, healthcare professionals’ knowledge and disease monitoring.

Patients generally felt that treating physicians and nurses provided sufficient attention to hand-related issues. During initial visits, nearly all patients received informational leaflets about hand problems. Additionally, national patient associations were a valuable source of information, and social media groups with peers provided practical advice and shared experiences. Most patients also independently sought information online. Despite these positive aspects, some patients noted room for improvement in delivering information. Educational materials, such as leaflets, were difficult to understand or not tailored to their situations. Patients strongly preferred face-to-face discussions that addressed their concerns, provided personalised guidance and allowed for questions.

Most information was provided in tertiary care centres, whereas patients felt that first- and second-line healthcare professionals often lacked sufficient information about SSc and associated hand problems. This lack of knowledge and understanding left some patients feeling unheard and contributed to delays in diagnosis. Physiotherapy was frequently perceived as unhelpful, with treatments described as insufficiently tailored to the specifics of SSc. In contrast, hand therapy was deemed more effective, as therapists demonstrated greater knowledge of hand-related issues and offered practical tips for daily life. Many patients also felt that healthcare professionals lacked sufficient knowledge regarding optimal treatment of hand function impairment in SSc. This knowledge gap often resulted in a reactive ‘wait-and-see’ approach rather than proactive management or timely referral. Patients emphasised that greater awareness and knowledge among all healthcare providers are essential to improving care.

While patients generally felt hand function was monitored and acknowledged by their treating physician, they noted that assessments were often generalised. They missed focusing on their hand function problems during regular clinical visits. Patients suggested that a more individualised approach, focused on specific complaints and needs, would significantly enhance care. Additionally, regular testing or assessments of hand function to monitor progression were suggested as a valuable improvement. This would lead to better understanding of the impairment and needs, and hopefully future treatment.

#### Management strategies

Patients managed their hand function impairment through various strategies, including contact with peers, exercises, tools and medication.

Many patients found comfort in exchanging stories and practical tips. Those peer contacts were found in patient organisations, during hospital visits and through social media. Others found it confronting and saddening to hear stories from others and preferred not to engage with others.

Patients commonly performed hand exercises at home, often without medical advice, sometimes with physical and hand therapists’ help. The results of the exercises differed; some experienced progress while many did not. Hand exercises seemed particularly helpful for stiffness complaints and maintaining function, while no long-term improvement is seen. Some patients also use connective tissue massage to counteract stiffness with a short-term positive effect. Swimming in warm water was further noted as helpful in reducing stiffness and keeping the hands flexible.

Different tools are used to help with tasks involving hands. Many patients used (heated) gloves to protect against cold, injuries and to keep ulcers clean. Specialised tools such as those with thicker handles were commonly used to provide better grip, including adjusted cutlery, writing utensils, bottle openers or tongs. Some patients found tools to help with (un)fastening buttons practical for dressing. Some preferred braces or (silver) splints for extra support or protection against bumping.

Different kinds of medications are prescribed with some effects on hand involvement. Furthermore, some patients use additional painkillers and moisturising cream. The effect of drugs was often challenging to describe, primarily because multiple medications are frequently used in conjunction.

## Discussion

This study highlights the substantial burden of hand function impairment in SSc and underscores the unmet need for targeted interventions. To our knowledge, this is the first international study to focus on patients’ perspectives of hand function impairment. Patients identified several determinants of hand dysfunction, reinforcing the multifactorial nature. Our findings confirm that hand function impairment is a key contributor to disability in SSc, significantly affecting daily activities, work participation and overall quality of life. Moreover, despite recognition of hand function loss as a significant challenge, current treatment strategies appear insufficient in addressing patients’ specific needs, with many reporting dissatisfaction related to available care options. These insights emphasize the urgent need for patient-centred approaches to improve hand function management in SSc.

The mentioned symptoms of hand function impairment highlight the extensive burden. All medical symptoms are previously described and often observed in clinical care [1]. Those medical symptoms often lead to the reported symptoms relating to hand function [3, 7, 17–22]. It was noticeable that patients frequently reported stress as a trigger for Raynaud, ulcer formation, puffy hands and infections. This finding aligns with previous research that stress can trigger a vasoconstrictive response and autoimmune activity [23–25]. The loss of sensation and numbness that many experience, even if there is no Raynaud attack, is not commonly described. This leads to patients being unable to feel what they are holding or doing, suggesting more neurological involvement than currently recognised and a need for further research [26]. Possible underlying mechanisms may include peripheral neuropathy, Raynaud and related chronic ischaemia, subcutaneous fibrosis or compression neuropathies such as carpal tunnel syndrome.

Handling small items and gripping objects were the most impacted tasks for patients, leading to difficulty with many activities requiring fine motor skills and necessitating support, resulting in frustration and sadness. Repetitive movements and cold environments frequently worsen those struggles. The reported impact of hand function symptoms corresponds with previous research on mental health, work ability, social participation and need for assistance [3, 6, 17, 27–30].

Although there were some positive experiences regarding hand function management, participants provided valuable points for improvement. Information, peer contact, tools, hand therapy, exercises and medication do not seem to be a one-size-fits-all solution due to the heterogeneity of the disease and personal preferences, so there is a need for a more personalised approach. Regular testing of the hand function would be a valuable improvement in monitoring progression for both patient and clinician. More knowledge is needed to provide personalised treatment and advice on which tools or therapy are helpful. A recently started longitudinal cohort study will hopefully provide information about the underlying risk mechanisms responsible for hand function impairment in SSc [31].

A strength of this study is the relatively large sample size for qualitative research [32]. Moreover, the SSc population is well represented in terms of gender, age and disease subset. We have included patients with different mRSS, mHAMIS and CHFS scores to assess different stages of impairment. Additionally, we included patients in two countries and did not see substantial response differences. A limitation of this study is that almost all participants are Caucasian, which reflects our study population but not the global SSc population. Additionally, patients were recruited from expert SSc centres with access to specialist nurses and specialized occupational therapists, meaning their experiences may not fully represent those treated in smaller secondary care hospitals. However, our findings suggest that even expert centres have room for improvement, highlighting the need for further evaluation of care across different healthcare settings. Furthermore, we did not obtain all CHFS scores due to a lack of response from participants.

Key implications for clinical practice and healthcare policy based on our findings are presented in Box 1.

In conclusion, we showed that patients are immensely impacted by limited hand function, which affects daily tasks, work, hobbies, psychological well-being and social activities. Tasks involving fine motor skills are especially troublesome. There is a substantial clinical need to better understand the underlying mechanisms and best approach to treat hand function impairment. A personalised approach with regular monitoring is preferred to address this unmet need.

## Supplementary material

Supplementary material is available at *Rheumatology* online.

## Supplementary Material

keaf476_Supplementary_Data

## Data Availability

The data underlying this article will be shared on reasonable request to the corresponding author.
