# Supplementary material for: Impact of hand function impairment on daily life of patients with systemic sclerosis: a qualitative study
Source: Rheumatology (Oxford). 2025 Sep 9;65(1):keaf476. doi: 10.1093/rheumatology/keaf476 (PMC12862391; doi:10.1093/rheumatology/keaf476)
Supplement: keaf476_Supplementary_Data [file keaf476_supplementary_data.zip › rhe-25-0732-File006.pdf]

Onderzoek naar ervaringen met handfunctiebeperkingen bij patiënten met systemische sclerose

INTERVIEW GUIDE (uitgebreide versie voor onderzoeker)

A INTRODUCTIE

Uitleg doel studie
In Nederland zijn ongeveer 4000 mensen met systemische sclerose (SSc). Een groot deel van de patiënten ontwikkelt klachten van de handen. Naar schatting heeft 90% van de mensen met SSc handfunctiebeperkingen, en 30 tot 50% ontwikkelt handcontracturen. Er is echter nog weinig bekend over de oorzaak, diagnostiek en behandeling. Beperkte handfunctie belemmert veel dagelijkse activiteiten. Beter begrip van en zicht op de impact van verminderde handfunctie kan patiënten, familieleden maar ook werkgevers helpen bij het omgaan met dagelijkse activiteiten en werk.

Wij willen graag van u horen of u klachten van de handen ervaart en wat de impact ervan op uw dagelijks leven is. Aanvullend willen we u vragen of u behoefte heeft aan betere zorg voor de klachten van de handen en of u daar ideeën over heeft.

Uitleg opname interview
Het geluid van het interview zal worden opgenomen, zodat we het achteraf kunnen terug luisteren en uitwerken.

Tekenen Informed consent

Demografische gegevens

Geslacht	man / vrouw / anders nl ..
Leeftijd	.. jaar
Opleidingsniveau	WO / HBO / MBO / middelbare school / anders nl ..
Gezinssituatie	vrijgezel / samenwonend / gehuwd / gescheiden / weduwe<br>.. kinderen (uitwonend / thuiswonend)
Werksituatie	betaalde baan (als ……………………………….., … uur/week)<br>Anders nl: uitkering / pensioen / vrijwilligerswerk / …

B INTERVIEW

1 Klachten handen

Klachten	Veel SSc patiënten hebben last van hun handen.<br>Wat zijn uw ervaringen op dit punt?<br><br>Aandachtspunten bij doorvragen:<br>o welke last precies? (bv. pijn, stijfheid, zwelling, krachtsverlies, gevoelsverlies, contractuur, Raynaud, wondjes, veranderd uiterlijk)<br>o welk onderdeel van de handen? (bv. vingertoppen, welke vingers, handrug, polsen)<br>o hoe lang geleden zijn de klachten begonnen? En hoe?<br>o hoe is het beloop over de tijd?

2 Impact van handfunctiebeperkingen

Impact	Wat voor impact hebben de klachten op uw dagelijks leven?<br><br>Vraag door naar de specifieke impact op de verschillende domeinen:<br><br>A Fysiek functioneren<br>Zoals ADL (wassen, aan/uitkleden/koken, schoonmaken), schrijven/typen, hobby/sport<br>• In welk opzicht wordt u fysieke functioneren beperkt door uw handen? (bv. activiteiten moeten aanpassen / stoppen en zo ja door welke klachten lukte het niet?<br><br>B Psychisch functioneren<br>• Welke gedachten en gevoelens roepen de klachten van de handen bij u op? (bv. verdriet, boosheid, angst, zorgen, somber)<br><br>C Sociaal functioneren<br>• In welk opzicht is uw thuissituatie beïnvloed door de klachten van de handen? (bv. voorbeelden waarin uw afhankelijk bent, verandering rolverdeling in gezin)<br>• In welk opzicht is uw werksituatie beïnvloed door de klachten van de handen? (bv. aanpassingen werk / minder werken)

3 Zorgbehoefte bij handfunctiebeperkingen

Zorgbehoefte	Een kwart van de patiënten met systemische sclerose geeft aan dat ze behoefte hebben aan betere zorg voor de klachten van de handen.<br>Wat zijn uw ervaringen op dit punt?<br><br>Aandachtspunten bij doorvragen:<br><br>A Informatievoorziening<br>• Wat voor informatie heeft u gehad over klachten van de handen bij deze ziekte en van wie?

	• Wat miste u aan informatie?<br><br>B Behandeling<br>• Welke dingen doet u zelf om de klachten te verminderen? (bv. pijnstilling, oefeningen, handschoenen)<br>• Welke behandelingen kent u voor de klachten van de handen? (bv. medicamenteus of paramedisch)<br>• Welke behandelingen heeft u zelf gevolgd voor de klachten van de handen? (bv. ergotherapie, fysiotherapie).<br>• Waarom heeft u deze behandeling gekozen, wat hield het precies in en wat waren de voor/nadelen? Zou u het aanraden aan anderen?<br>• Heeft u ideeën over hoe de behandeling van klachten van de handen kan worden verbeterd? Waar heeft u behoefte aan?

5. Afsluitende vraag

Zijn er nog zaken die niet aan bod zijn gekomen in het interview die u graag zou willen benoemen?

Afronding

- Maak zichtbaar dat het interview is beëindigd door de geluidsopname te beëindigen.
- Stel de deelnemer in de gelegenheid te reflecteren.
- Wijs de deelnemer op de mogelijkheid dat wanneer hij/zij, als gevolg van het interview, vragen heeft hij/zij contact met de interviewer kan opnemen.
