# Supplementary material for: Impact of hand function impairment on daily life of patients with systemic sclerosis: a qualitative study
Source: Rheumatology (Oxford). 2025 Sep 9;65(1):keaf476. doi: 10.1093/rheumatology/keaf476 (PMC12862391; doi:10.1093/rheumatology/keaf476)
Supplement: keaf476_Supplementary_Data [file keaf476_supplementary_data.zip › rhe-25-0732-File007.pdf]

EXPERIENCE OF HAND FUNCTION LIMITATIONS IN PATIENTS WITH SYSTEMIC SCLEROSIS

HANDSOME Experience study
Interview guide
v1.0 05-07-2023

Introduction
It has been estimated that up to 90% of people with systemic sclerosis have limitations in the use of their hands and around 30-50% develop hand contractures.
We want to establish how the effects of reduced hand function and/ or contractures on the day to day lives of patients. A better understanding of this will 1) help patients and family members in their day to day planning and management of activities; 2) guide clinicians in the management of patients with hand impairment.

During the interview we will first explore the course of the patient's scleroderma and the onset and course of hand function limitations. We will then establish the impact of hand function limitations followed by unmet needs in care and patient education with regard to hand impairment.

1 Disease course

Onset	• Describe the onset of the symptoms you experienced in relation to your systemic sclerosis?<br>• What were your first symptoms of the disease?<br>• How long after your diagnosis did you experience symptoms in relation to your hands/ fingers? What symptoms did you experience?<br><br>*Points to consider when asking:*<br>• Ask for more detail on symptoms (e.g., pain, stiffness, swelling, loss of strength, pins and needles, tingling, numbness, contracture, Raynaud's, wounds, skin, joints, tendons, altered appearance)<br>• Which part of the hands? (e.g. fingertips, which fingers, back of hand, wrists)
Course	• How did your hand symptoms progress?<br>o Have you been given any treatments to improve your hand symptoms?<br>o What was the effect of the treatment(s)?<br>o Did the symptoms change? Are they worse/better during day/night, during activity/at rest, same every day/changing?

2 Impact of hand function impairment

Impact	• In what ways are you limited by your hands in your daily life?<br>o Daily activities (washing, dressing/cooking, cleaning), writing/typing, hobby/sports<br>o Have you had to adjust or discontinue certain activities? If so, what made it impossible (pain, loss of strength, loss of sensation?)

	• Do you have a paid job? Or do you do volunteer work?<br>○ In what ways are you limited by your hands during your work activities? (e.g. work adjustments / working less)<br><br>• In what ways do the hand function limitations impact your mental health? (e.g. feelings of sadness, anger, fear)<br>• In what ways does the appearance of your hands impact your mental health? (e.g. feelings of sadness, shame)<br><br>• In what ways do hand function limitations impact your relationships with others? (e.g., examples in which you are dependent and change of roles within family)
	• Where do you find support in dealing with these limitations? (Family/ Patient Association/ Internet/ healthcare professionals)<br><br>• Is your doctor or nurse specialist/company doctor/employer paying attention to these limitations?

3 Management of hand function impairment

Education	• Have you received or found information about how systemic sclerosis affects hand function? By whom?<br>• What were you offered in terms of information, what sources of information did you use?<br>• Was the information you received about hand function in systemic sclerosis complete, was it easy to understand?
Treatment	• Do you do anything for yourself to try and reduce symptoms? (e.g. pain relief, exercises, gloves)<br>• Have you received/do you use non-pharma treatments for your reduced hand function? If so, what? e.g. occupational therapy, physical therapy).<br>• Why did you choose this treatment, what exactly did it involve and what were the effects and side effects?<br>• What would you change about your treatment looking back on it?
Expectations	• What is your expectation for the future regarding your hand function (improving/deteriorating/stable)<br>• How does this make you feel?<br>• Are your expectations and concerns adequately addressed?
Unmet needs	• What are areas of improvement for care/information/guidance for your hand function limitations?

Other issues

Are there any issues not covered in the interview that you would like to mention?

Closure

- Make clear that the interview has ended by ending the audio recording.
- Provide an opportunity for the participant to reflect.

- Point out to participants that if, as a result of the interview, they have questions or otherwise experience difficulties they can contact the interviewer or their doctor.
